# Transient Fetal Nonimmune 2:1 Atrioventricular Block Possibly Secondary to Cannabis Exposure

**DOI:** 10.1016/j.case.2026.03.010

**Published:** 2026-05-26

**Authors:** Ananya Chandra, Sandra Waller, Ron Wakai, Victor Suarez, Janette Strasburger, Matthew L. Moehlmann

**Affiliations:** a Department of Pediatrics, University of Chicago, Chicago, Illinois; b Advocate Good Samaritan Hospital, Downers Grove, Illinois; c Department of Physics, University of Wisconsin–Madison, Madison, Wisconsin; d AdventHealth Medical Center, Hinsdale, Illinois; e Children's Wisconsin, Milwaukee, Wisconsin

**Keywords:** Fetal bradyarrhythmias, Fetal atrioventricular block, Non-immune mediated, Cannabis exposure

## Abstract

• Transient AV block in utero is usually from structural or autoimmune causes.
• Studies show cannabis crosses the placenta and affects cardiac function and rhythm.
• Transient AV block from cannabis has been observed in adolescents and adults.
• Consider cannabis as a cause of fetal AV block after excluding other etiologies.

## Graphical abstract

**Figure:**
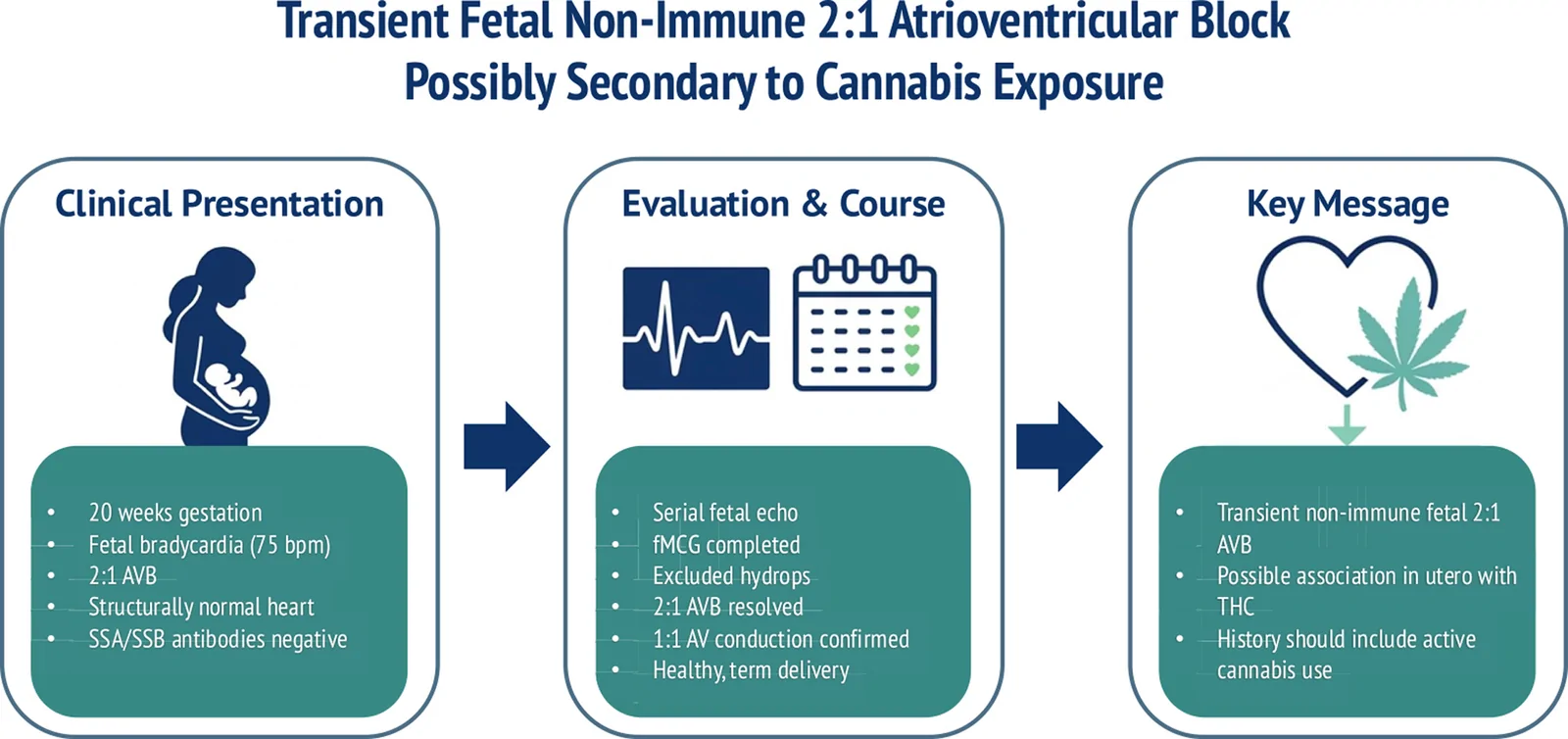

## Introduction

Fetal atrioventricular block (AVB) is an uncommon fetal arrhythmia, often secondary to structural congenital heart disease or maternal autoimmune conditions. Fetal echocardiography is the primary modality for diagnosing arrhythmias in fetal life. We present an unusual case of nonimmune mediated fetal AVB that was diagnosed with fetal echocardiography, possibly secondary to cannabis exposure.

## Case Presentation

A 31-year-old gravida 2 para 0 was referred for fetal echocardiography at 20 weeks' gestation due to fetal bradycardia of 75 beats per minute (bpm) at maternal fetal medicine clinic. Fetal heart rate had been 138 bpm earlier that day during a scan with the obstetrician. There was no significant maternal history of arrhythmias, medication use, or suspicion for infection. There was also no family history of long QT syndrome or rheumatologic conditions. On presentation, the child's fetal echocardiogram demonstrated a structurally normal heart with normal systolic function (Figure 1, Video 1). However, there was significant bradycardia, with a heart rate of 75 bpm. When analyzing the rhythm further, the child was in a 2:1 rhythm with a regular atrial rate of 150 bpm and ventricular rate of 75 bpm (Video 1). This was seen by M-mode (Figure 2) and spectral Doppler of ductus venosus, left ventricular inflow/outflow, and pulmonary venous Doppler (Figure 3). The PR interval was normal at 120 milliseconds, and there were no premature atrial contractions. Additionally, there was no pericardial effusion, endocardial fibroelastosis, tricuspid regurgitation, or mitral regurgitation (Figure 4, Video 2). At this time, the mother had further laboratory testing including a complete blood count, electrolytes, thyroid function testing, and anti-Sjogren's syndrome A (SSA) and anti-Sjogren's syndrome B (SSB) antibodies, all of which ultimately returned normal. Their 25-hydroxy vitamin D level was less than 30 ng/mL. The patient did report regular marijuana use during pregnancy and testing for tetrahydrocannabinol (THC) was positive. The patient denied using other recreational substances, and drug screen was otherwise negative.

**Figure 1:**
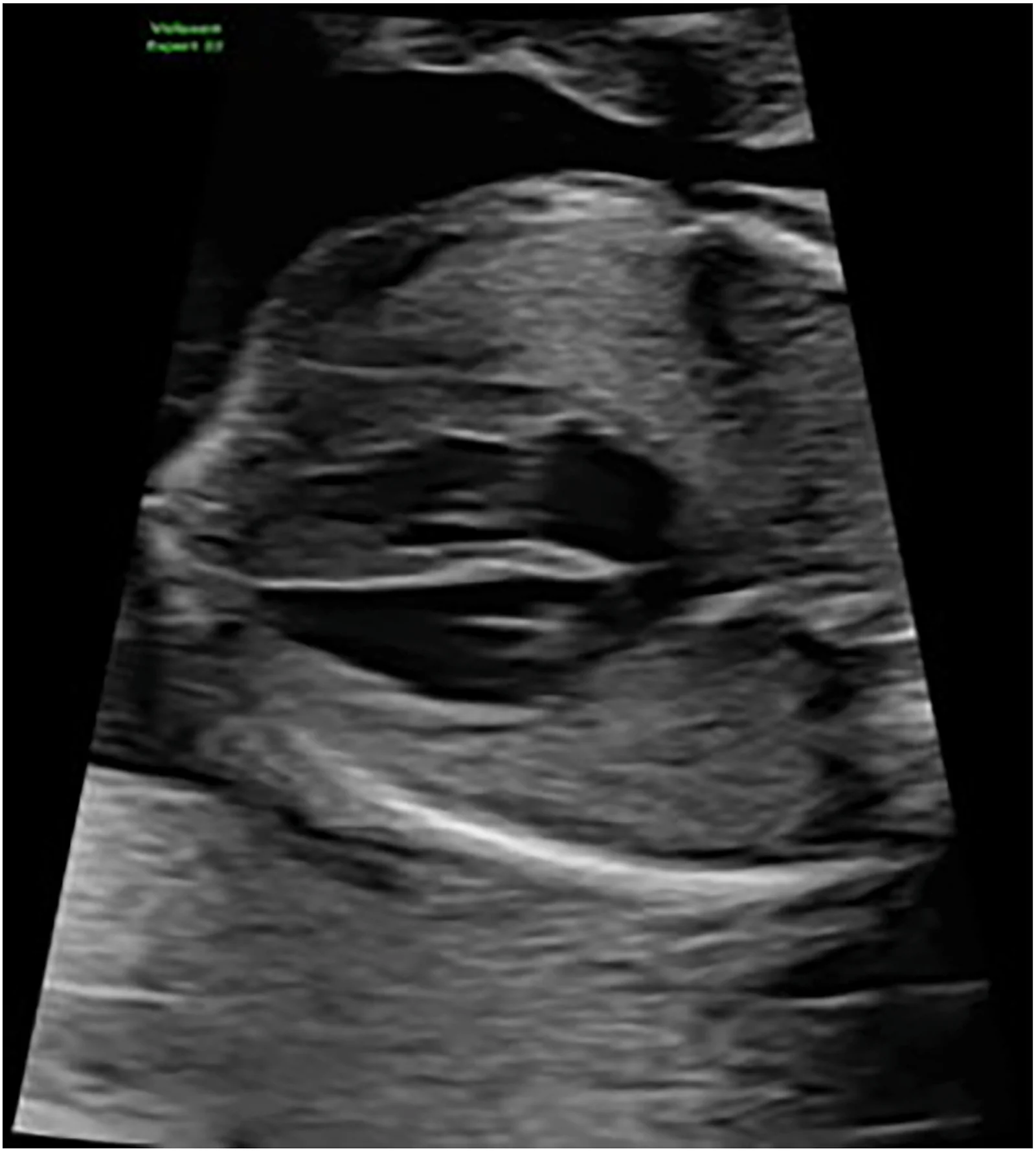
Two-dimensional fetal echocardiography, 4-chamber view, demonstrates the fetal heart with normal fetal anatomy, no pericardial effusion, and no endocardial fibroelastosis.

**Figure 2:**
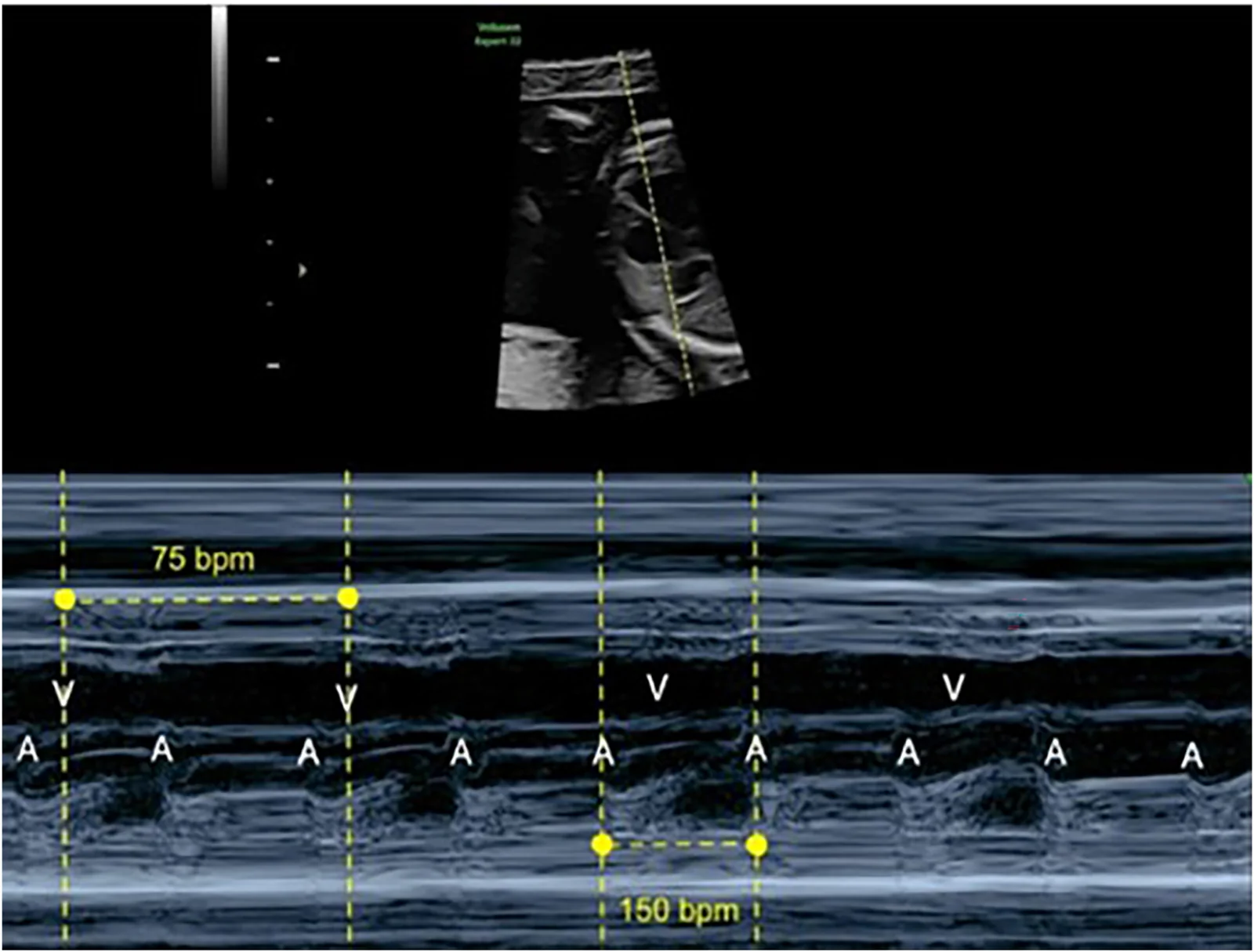
Two-dimensional fetal echocardiography, 4-chamber view with M-mode display, demonstrates ventricular rate (V) bradycardia (75 bpm) and normal atrial (A) rate (150 bpm) with 2:1 AVB.

**Figure 3:**
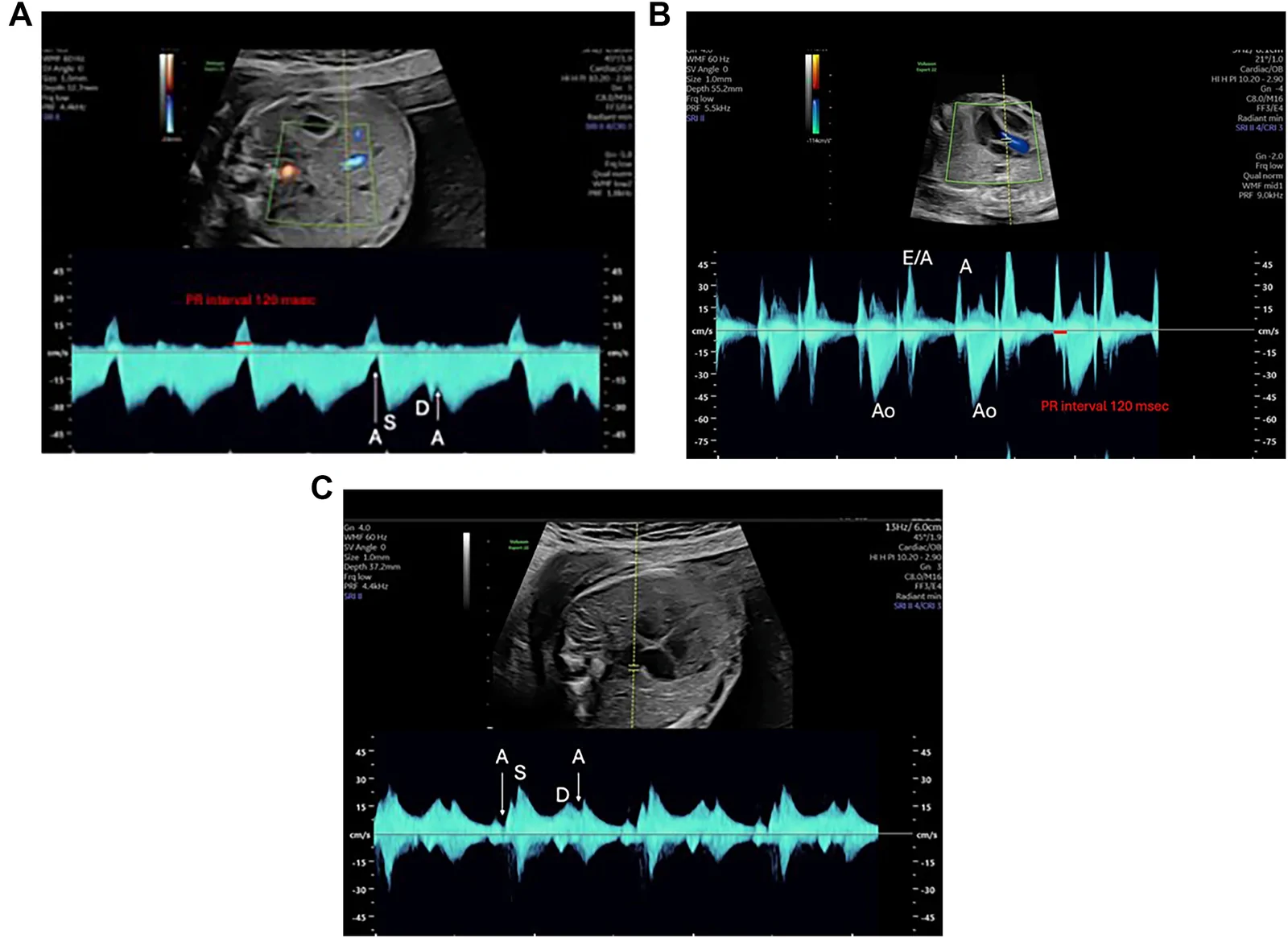
Two-dimensional fetal echocardiography, spectral Doppler display of the ductus **(A)**, mitral inflow and aortic outflow **(B)**, and pulmonary vein flow **(C)** during bradycardia with 2:1 AVB, demonstrates A-wave reversal with normal PR interval of 120 milliseconds, an A-to-A interval of 150 bpm, and the 2:1 relationship of the A wave with the ventricular systolic (S) and diastolic (D) waves. *A*, Atrial systole; *Ao*, aortic ejection; *D*, ventricular diastole; *E/A*, superimposition of early diastolic filling and atrial systole; *S*, ventricular systole.

**Figure 4:**
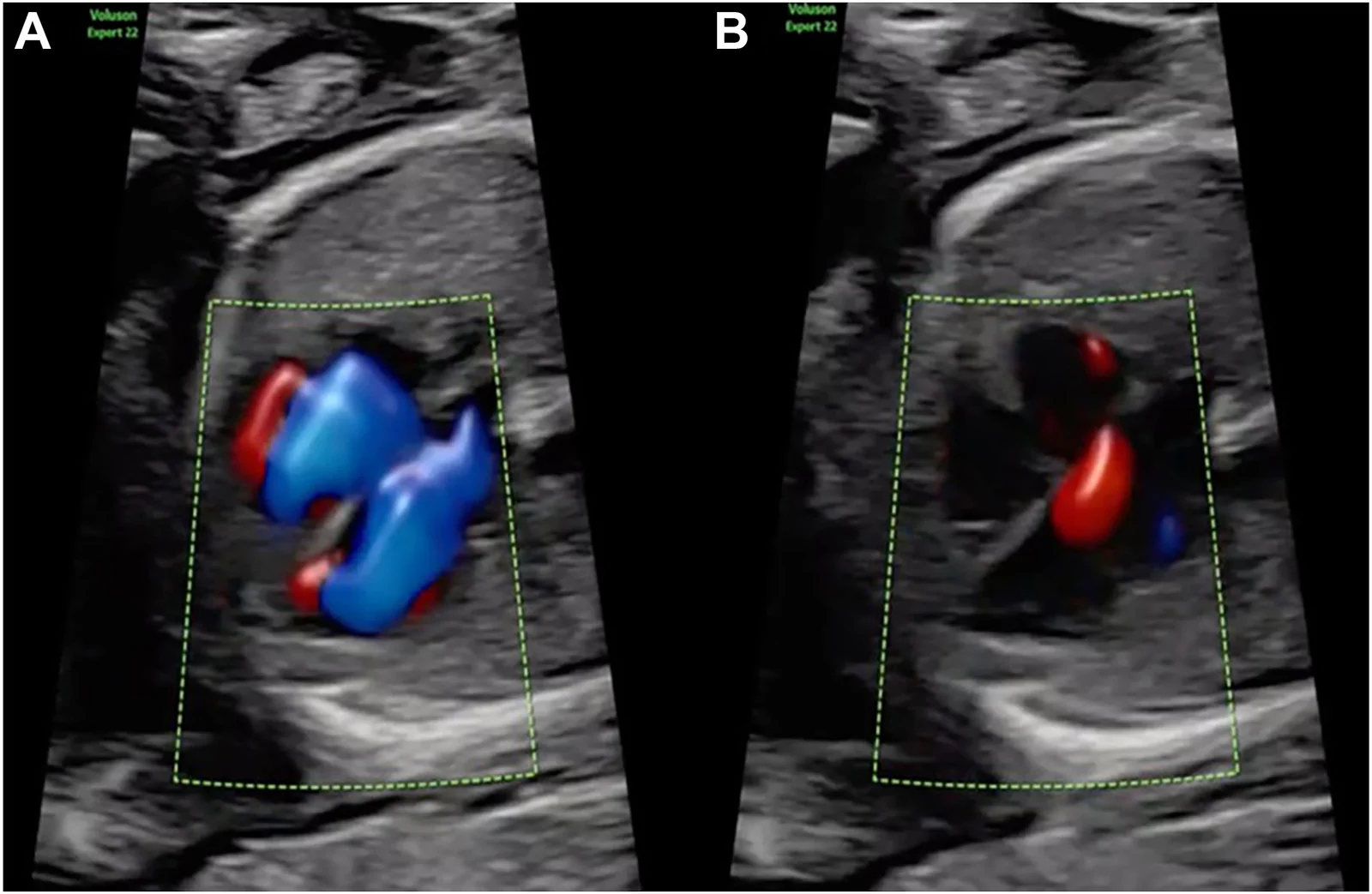
Two-dimensional fetal echocardiography with color-flow Doppler, 4-chamber view in diastole **(A)** and systole **(B)**, demonstrates no mitral or tricuspid valve regurgitation and normal laminar flow (*red* in panel B) in the left ventricular outflow tract during bradycardia with 2:1 AVB.

While immune-mediated AVB was on the differential, we did not start any anti-inflammatory therapies such as steroids or intravenous immunoglobulin due to reassuring echocardiographic findings. A plan was made for weekly fetal echocardiograms, closer monitoring with maternal fetal medicine and obstetric providers, and obtaining parental electrocardiograms (ECGs). The mother agreed to stop using THC for the duration of the pregnancy (but repeat testing did not occur).

At the 21-week visit, fetal echocardiogram demonstrated a period of 1:1 fetal atrioventricular (AV) conduction (Video 3) before reverting to a pattern of bradycardia with 2:1 AVB. Again, the heart appeared structurally normal. The maternal ECG demonstrated normal sinus rhythm with normal QT interval. At 22 weeks, the fetus maintained a 1:1 rhythm and there were no other reports of fetal bradycardia of AVB. At this point, a fetal magnetocardiography (fMCG) study was obtained, which demonstrated >5 decelerations down to 100 bpm (Figure 5). This resolved without inducing AVB, suggesting a normal AV conduction. The signal-averaged fMCG QTc interval was 338 milliseconds (Figure 6). The child was ultimately delivered at term via normal spontaneous vaginal birth. Initial ECG on day of life 1 demonstrated a prolonged QT interval of 473 milliseconds. Neonatal toxicology screen was not performed and is not routinely performed in neonates. At 2 weeks of life, the QT interval normalized to 447 milliseconds. The baby continued to have normal weight gain, regular heart rate, and normal ECG and was subsequently discharged from the pediatric cardiology clinic.

**Figure 5:**
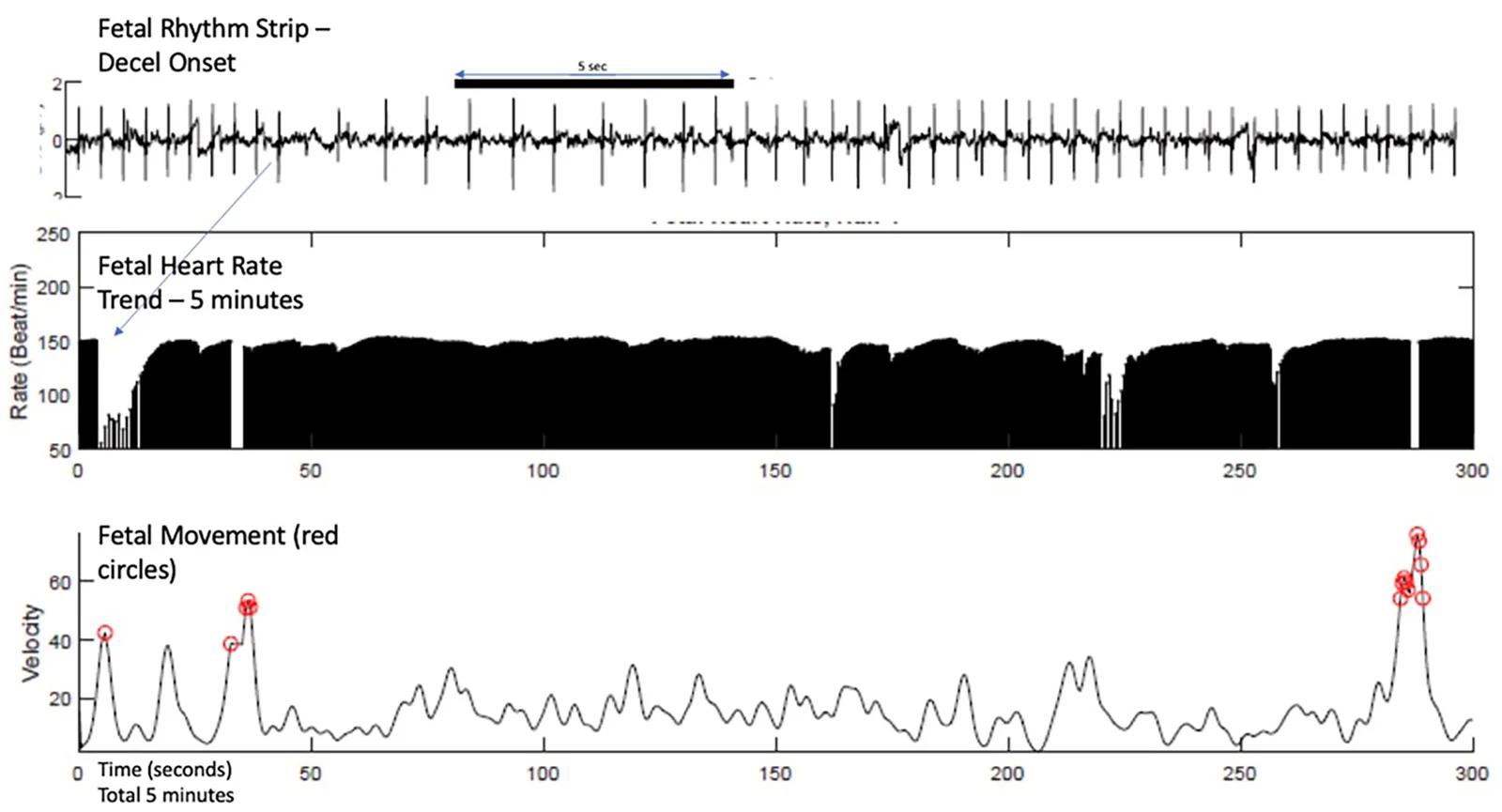
Fetal magnetocardiography of the fetal heart rate, obtained after resolution of the bradycardia and the 2:1 AVB (22 weeks), displayed over 5 minutes (0-300 seconds), demonstrates that the fetus had 5 transient decelerations (*middle row*; HR < 100 bpm); a single lead rhythm strip *(top row)* from a transient deceleration starting at 9 seconds is likely associated with hypervagatonia induced by brisk fetal movement *(lower row, red circles)*; AVB was not triggered by the hypervagatonia and is an indication that the AV conduction has successfully recovered.

**Figure 6:**
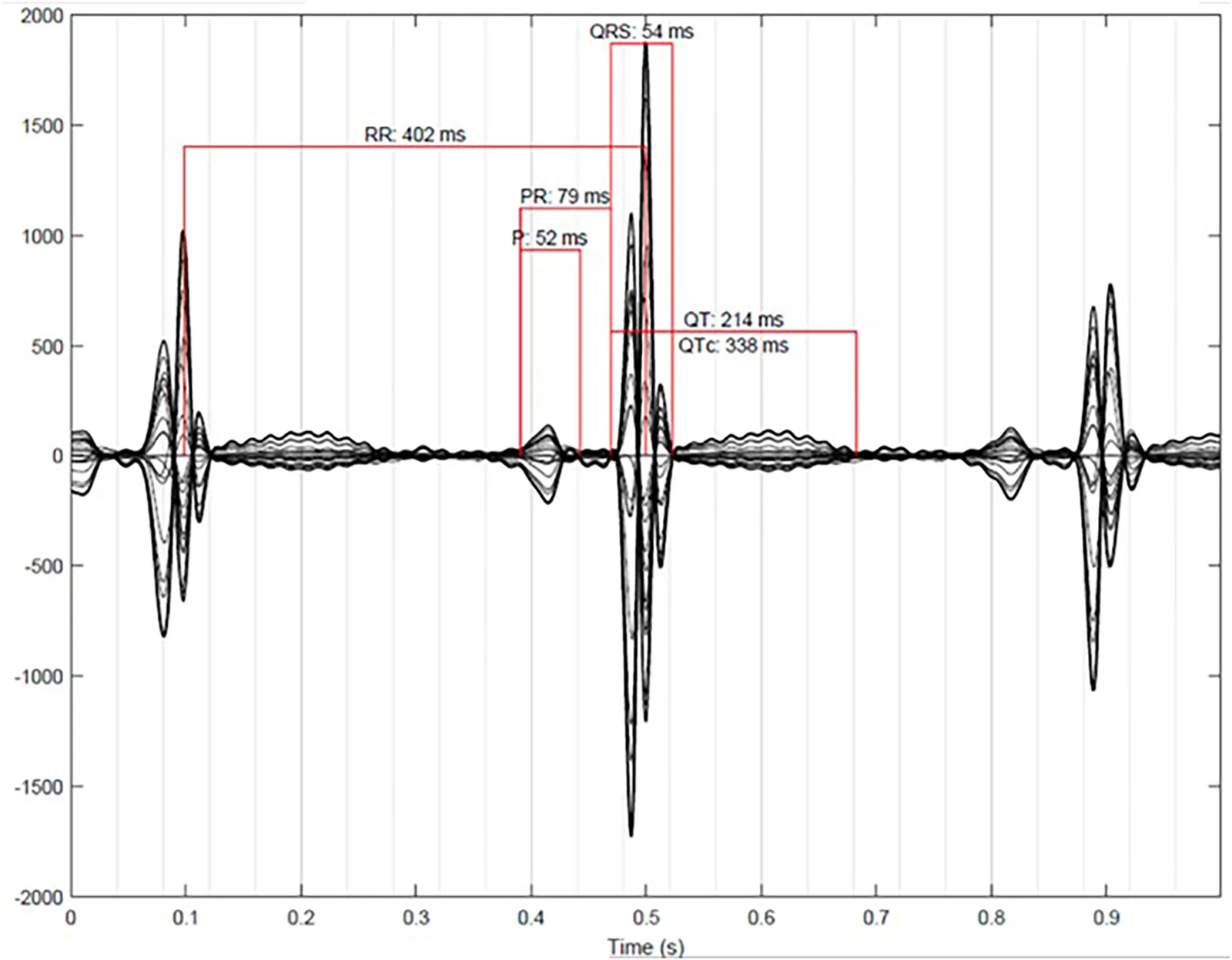
Fetal magnetocardiography signal-averaged display, obtained after resolution of the bradycardia and the 2:1 AVB (22 weeks), averaged from 20 seconds, demonstrates a normal PR, normal QTc (338 milliseconds), and normal other cardiac time intervals.

## Discussion

Fetal arrhythmias are broadly categorized into premature contractions, tachyarrhythmias, and bradyarrhythmias.^1^ Bradyarrhythmias include sinus bradycardia, fetal AVB, blocked atrial bigeminy, and congenital long QT syndrome and represent a relatively common abnormality encountered in fetal cardiology. Accurate identification of underlying etiology is essential, as these conditions vary widely in prognosis and management.

Fetal AVB is a disturbance of communication between the atria and ventricles, either through congenital displacement or inflammatory damage of the sinoatrial node. Approximately 45% of fetal AVB is associated with congenital heart defects, while nearly 50% is related to autoimmune disease.^2^^,^^3^ The remaining 5% occurs in isolation, such as transient nonimmune AVB. Fetal AVB has been reported anywhere from 17 to 30 weeks’ gestation and can result in developmental delay or even fetal demise if progression to complete AVB occurs.^3^^,^^4^ Consequently, careful rhythm surveillance during fetal life, particularly in the second trimester, is critical.

In maternal autoimmune disease, one of the most common etiologies of fetal AVB, anti-SSA (Ro) and anti-SSB (La) antibodies cross the placenta and directly affect the fetal cardiac conduction system by promoting inflammation and fibrosis of the AV node, leading to varying degrees of AVB.^2^^,^^3^ Therefore, maternal serologic testing is an important component of diagnostic evaluation. In our case, the mother tested negative for these autoantibodies, suggesting a nonimmune etiology for the AVB. Our patient also did not have findings for structural heart disease (such as congenitally corrected transposition of the great arteries, heterotaxy, or left atrial isomerism) or signs of fetal compromise on echocardiogram. Other more common differentials, such as blocked atrial bigeminy and fetal long QT syndrome, also seemed unlikely without family history of arrhythmias.

Management of suspected autoimmune-mediated AVB typically involves transplacental treatment with steroids and/or intravenous immunoglobulin.^1^^,^^5^ In this case, maternal therapy was not indicated because the diagnosis of immune-mediated AVB was not clear. Specifically, there were no echocardiographic findings suggestive of conduction disease, AV valve regurgitation, prolonged PR interval, pericardial effusion, or endocardial fibroelastosis. Furthermore, rapid turnaround for maternal SSA/SSB levels at our institution allowed for prompt clarification of etiology. This resulted in the diagnosis of transient nonimmune fetal AVB.

Although the literature on this topic remains sparse, there have been rare reports of transient AVB in fetuses with otherwise normal evaluation.^6^ While there are previously documented cases of isolated nonimmune fetal AVB without structural heart disease or long QT syndrome in the literature, there have not been much data to indicate further causation for these abnormalities.^6^^,^^7^ The literature does show that outcomes of 60% of fetuses with nonimmune mediated second-degree AVB were favorable, while other cases resulted in hydrops or permanent pacing.^8^

Notably, the maternal history in our patient revealed regular cannabis use during pregnancy before diagnosis. After all testing was completed, we were left with the history of cannabis use and AVB. Prior reports of AVB in adolescents and adults associated with marijuana use prompted consideration of its role in the 2:1 AVB in this child.

The temporal association between ongoing marijuana exposure and development of the AVB raised the question of whether THC, the psychoactive component of cannabis, may have contributed to the observed rhythm abnormality. Animal studies and limited human data suggest that cannabis can cross the placental barrier and has the potential to affect fetal development, including cardiovascular function.^9^^,^^10^ THC is known to enhance vagal tone, particularly with repetitive use.^11^ There is also emerging evidence that cannabinoids may interact with cardiac ion channels and autonomic regulation, possibly leading to rhythm disturbances such as bradyarrhythmias and AVB.^12^^,^^13^ We speculate that the marijuana use created high vagal tone in the fetus (which was noted on fMCG study), and this gradually improved after counseling the patient that this may be a contributing factor.

While the fetal autonomic nervous system remains immature at 20 weeks of gestational age, dysregulation of the early autonomic nervous system could potentially explain the more frequent deep physiological decelerations in this fetus when compared to normal fetal decelerations. A recent study of adults showed that the autonomic dysregulation further exacerbated arrhythmia risk.^14^ Receptor activation by THC enhanced sympathetic activity and increased heart rate and susceptibility to tachyarrhythmias, while vagal suppression reduced parasympathetic tone.^15^ In some cases, THC-induced parasympathetic rebound resulted in bradyarrhythmias or AVB. In nearly all cases, the AVB was in younger subjects and those without underlying cardiovascular disease.^15^

Although we did not consider the differences between synthetic and nonsynthetic marijuana in this patient, this is an additional consideration when assessing possible marijuana-associated arrhythmias. There are some reported cases of severe bradycardia and hypotension from synthetic marijuana in adults and adolescents.^16^^,^^17^ These substances are more difficult to test for than nonsynthetic marijuana, and more detailed laboratory testing would need to be done if these substances were being considered.

While we cannot definitively establish causation, the mother's marijuana use raises the possibility of an association between in utero THC exposure and transient fetal AVB. There are no known reported cases in the literature describing any temporal relation with THC use and AVB, making this a unique consideration. This case underscores the need for further research into the cardiac effects of cannabis during pregnancy and highlights the importance of including THC exposure in the differential diagnosis of nonimmune fetal AVB. As cannabis use becomes more widespread, clinicians should maintain a high index of suspicion and counsel patients accordingly.

## Conclusion

Cannabis exposure should be considered as a possible cause of nonimmune mediated AVB in fetal life. Clinicians should maintain a high index of suspicion for THC exposure when working up nonimmune mediated causes of fetal AVB, although further research is needed to establish causation.

## Consent Statement

The authors declare that since this was a non-interventional, retrospective, observational study utilizing de-identified data, informed consent was not required from the patient under an IRB exemption status.

## Ethics Statement

The authors declare that the work described has been carried out in accordance with The Code of Ethics of the World Medical Association (Declaration of Helsinki) for experiments involving humans.

## Funding

Funding for this paper was provided by 10.13039/100000002NIH R01HL063174 to Wakai and 10.13039/100000002NIH R01HL143485 to Strasburger.

## Disclosure Statement

The authors reported no actual or potential conflicts of interest relative to this document.

## Declaration of generative AI and AI-assisted technologies in the manuscript preparation process

During the preparation of this work the authors used Perplexity AI in order to assist with creation of the graphical abstract. After using this tool, the authors reviewed and edited the content as needed and take full responsibility for the content of the published article.

## Supplementary Data

Video 1 Two-dimensional fetal echocardiography, 4-chamber view, demonstrates the fetal heart with normal fetal anatomy, no pericardial effusion, no endocardial fibroelastosis, and bradycardia with 2:1 AVB.

Video 2 Two-dimensional fetal echocardiography with color-flow Doppler, 4-chamber view, demonstrates no mitral or tricuspid valve regurgitation and normal laminar flow in the left ventricular outflow tract during bradycardia with 2:1 AVB.

Video 3 Two-dimensional fetal echocardiography, 4-chamber view, demonstrates the fetal heart with normal heart rate with 1:1 AV conduction.
